# Emerging Role of Gut Microbiota in Modulating Response to Therapies in IBD

**DOI:** 10.3390/microorganisms14051082

**Published:** 2026-05-11

**Authors:** Bianca Bartocci, Angelo Del Gaudio, Marco Murgiano, Alfredo Papa, Giovanni Cammarota, Antonio Gasbarrini, Franco Scaldaferri, Loris Riccardo Lopetuso

**Affiliations:** 1 Dipartimento di Medicina e Chirurgia Traslazionale, Università Cattolica del Sacro Cuore, 00168 Rome, Italy; biancabartocci97@gmail.com (B.B.); delgaudioangelo@gmail.com (A.D.G.); marcomurgiano1@gmail.com (M.M.); alfredo.papa@unicatt.it (A.P.); giovanni.cammarota@unicatt.it (G.C.); antonio.gasbarrini@unicatt.it (A.G.); franco.scaldaferri@unicatt.it (F.S.); 2 Medicina Interna e Gastroenterologia, CEMAD Centro Malattie dell’Apparato Digerente, Dipartimento di Scienze Mediche e Chirurgiche, Fondazione Policlinico Universitario Gemelli IRCCS, 00168 Rome, Italy; 3 Dipartimento di Scienze della Vita, della Salute e delle Professioni Sanitarie, Università degli Studi Link, 00165 Rome, Italy

**Keywords:** inflammatory bowel disease, gut microbiota, dysbiosis, biologic therapy, small molecules, metabolomics, therapeutic response, precision medicine

## Abstract

The gut microbiota is increasingly recognized as a key contributor in the pathogenesis and progression of inflammatory bowel disease (IBD). Compared with healthy individuals, patients with IBD show marked dysbiosis, characterized by reduced microbial diversity, an expansion of facultative anaerobes such as Proteobacteria, and a depletion of obligate anaerobes within the Firmicutes phylum. These changes have been implicated in the perpetuation of intestinal inflammation, disruption of mucosal immune homeostasis, and altered metabolic functions, further underscoring the microbiota’s relevance in IBD pathophysiology. However, microbiota-driven insights have not yet been consistently translated into therapeutic stratification or clinical decision-making. A major challenge lies in the complex and dynamic interplay between the gut microbiota and various treatment modalities, including conventional immunosuppressants, biologics, and small-molecule inhibitors. While accumulating evidence suggests that IBD treatments may modulate microbial composition and function, it remains unclear whether these changes represent a direct pharmacological effect or are secondary to inflammation control. Additionally, there is a lack of comparative data on microbiota profiles associated with differential responses to various therapeutic classes, limiting the implementation of microbiota-informed precision medicine. In this review, we synthesize current evidence on the association between gut microbiota composition and treatment outcomes, focusing on biologic agents and small-molecule therapies. Furthermore, we discuss the potential of microbiota-targeted strategies, such as fecal microbiota transplantation (FMT) and precision probiotics, in enhancing therapeutic response. A deeper understanding of host–microbe interactions could enable a more personalized and effective approach to IBD management.

## 1. Introduction

Inflammatory bowel diseases, including Crohn’s disease (CD) and Ulcerative Colitis (UC), are chronic and relapsing disorders whose pathogenesis is still unclear [1]. The most accepted hypothesis is that it is a genetically determined condition influenced by environmental and socioeconomic factors such as lifestyle, diet, and antibiotic use [2].

A critical aspect of IBD pathogenesis involves the intestinal microbiota, which plays a pivotal role in disease onset, progression, and chronicity. However, it remains unresolved whether dysbiosis—a significant alteration in the intestinal microbiota—is a cause or consequence of IBD [3]. Both CD and UC are consistently associated with dysbiosis, characterized by a decrease in microbial species richness, commonly referred to as reduced alpha diversity.

The microbiota itself, containing at least 100 times more genes than the human genome, is highly modifiable by external factors, such as diet, antibiotic exposure, life events, and infections [4,5].

In the small intestine, the concentration of microorganisms ranges from 10^3^ to 10^9^ cells per gram, while in the colon, bacteria can reach a concentration of up to 10^12^ cells per gram of luminal contents [4,6].

In particular, the human microbiota of a healthy individual is composed of four main bacterial phyla: Firmicutes, Bacteroidetes, Proteobacteria, and Actinobacteria [7], with the first two accounting for the largest proportion. IBDs are characterized by a decrease in microbial species richness, known as alpha diversity [8].

Several studies have demonstrated differences in microbial composition among patients with CD, UC, and non-IBD control subjects: IBD patients are characterized by lower microbial diversity and increased growth of facultative anaerobes such as Proteobacteria and reductions in obligate anaerobes such as Firmicutes [9].

Nowadays, there is a wide selection of treatments that is still steadily increasing: steroids, anti-inflammatories, and biological drugs.

Despite that, unfortunately, only a small percentage of patients achieve sustained response. This may be due to the lack of established sequential guidelines for different therapeutic classes in the case of failure of previous therapy. Furthermore, the absence of predictive markers to determine the most suitable medication for the patient delays the time to achieve response and disease remission [10].

In this context, despite the increasingly recognized role of the intestinal microbiota in the pathogenesis of IBD, there is still insufficient data on the microbiome as a predictive marker of treatment response. This review aims to examine the available literature on the bidirectional relationship between the gut microbiota and therapeutic interventions in inflammatory bowel disease, with the objectives of:

(i) Identifying which microbial features are consistently associated with therapeutic response;
(ii) Determining which therapies plausibly interact with gut microbiota through direct metabolic effects as opposed to indirect mechanisms mediated by inflammation control;
(iii) Evaluating which microbiota-derived markers are currently closest to clinical applicability for guiding therapeutic decision-making.

## 2. Materials and Methods

This narrative review was conducted to summarize and critically appraise current evidence on the role of the gut microbiota in modulating therapeutic response in inflammatory bowel disease (IBD).

### 2.1. Literature Search Strategy

A comprehensive literature search was performed in the PubMed/MEDLINE, Embase, and Scopus databases to identify relevant articles published between January 2000 and June 2025. The search strategy combined free-text terms and Medical Subject Headings (MeSH) related to IBD, gut microbiota, therapeutic interventions, and treatment outcomes. Search terms included, but were not limited to: *“inflammatory bowel disease,” “Crohn’s disease,” “ulcerative colitis,” “gut microbiota,” “microbiome,” “dysbiosis,” “metagenomics,” “metabolomics,” “microbial metabolites,” “biologic therapies,” “anti-TNF,” “vedolizumab,” “ustekinumab,” “anti-IL-23,” “JAK inhibitors,” “small molecules,” “thiopurines,” “mesalamine,” “corticosteroids,” “fecal microbiota transplantation,” “probiotics,” “treatment response,” “clinical remission,” “endoscopic remission,” and “loss of response.”

### 2.2. Study Selection and Eligibility Criteria

Eligible studies included original clinical research articles, randomized controlled trials, prospective and retrospective observational studies, and translational studies evaluating the association between gut microbiota composition, microbial metabolites, or microbiome-derived signatures and therapeutic outcomes in patients with IBD. Studies assessing both fecal and mucosal microbiota were considered. To reduce selection bias and ensure the quality of included evidence, study selection was guided by predefined eligibility criteria. Priority was given to studies reporting clearly defined therapeutic outcomes alongside microbiota characterization. Studies were critically appraised for sample size, methodological rigor, and clarity of outcome reporting. Exclusion criteria comprised case reports, editorials, conference abstracts, non-peer-reviewed articles, and publications not written in English. Review articles were not included in the qualitative synthesis but were used to identify additional primary studies.

A total of approximately 214 articles were initially identified in the literature search. After removal of 54 duplicates, 160 articles were screened for relevance, and 102 full-text articles were finally included in the narrative synthesis.

### 2.3. Data Synthesis

Given the substantial heterogeneity across studies in terms of patient populations, therapeutic classes, microbiota assessment techniques (including 16S rRNA gene sequencing, shotgun metagenomics, and metabolomics), and outcome definitions, a quantitative synthesis or meta-analysis was not feasible. Therefore, findings were synthesized qualitatively and organized according to therapeutic class and type of microbiota-related outcome.

Definitions of treatment response—including clinical, endoscopic, and biomarker-based endpoints—were accepted as reported in the original studies, and this variability was considered when interpreting results.

**Methodological Considerations:** This review follows a narrative format and was not registered or conducted according to formal PRISMA guidelines. While this approach may be associated with selection bias and limit the strength of the conclusions, it allows the integration of diverse lines of evidence, provides a comprehensive conceptual overview, and highlights emerging trends and knowledge gaps. This format was chosen to offer a broad synthesis of the current literature and to inform future research directions in gut microbiota-mediated modulation of therapeutic response in IBD.

## 3. Gut Microbiota in IBD

The intestinal microbiota in patients with IBD is profoundly altered in terms of diversity, composition, and functional activity, a condition commonly referred to as gut dysbiosis. Compared with healthy controls, patients with Crohn’s disease and Ulcerative Colitis consistently display reduced microbial diversity, depletion of obligate anaerobes—particularly within the Firmicutes phylum—and expansion of facultative anaerobes such as Proteobacteria, including members of the Enterobacteriaceae family [9,10,11,12].

Several pro-inflammatory taxa have been implicated in IBD pathogenesis, including adherent-invasive *Escherichia coli*, *Ruminococcus gnavus*, and *Fusobacterium*, whereas bacteria with recognized anti-inflammatory properties, such as *Faecalibacterium prausnitzii*, are markedly reduced, particularly in ileal Crohn’s disease [13,14,15]. This imbalance is associated with impaired production of short-chain fatty acids and disruption of mucosal immune homeostasis.

Alterations in microbiota composition have also been linked to disease activity. Reduced alpha diversity correlates with increased inflammatory burden, whereas lower fecal calprotectin levels—particularly in pediatric populations—are associated with higher microbial diversity [16,17,18]. In addition, active disease is characterized by enrichment of pathobionts, including *Escherichia coli* strains with adherent-invasive properties, further supporting the contribution of dysbiosis to intestinal inflammation [19,20,21].

Beyond bacteria, IBD-associated dysbiosis also involves other microbial kingdoms. Increased abundance of fungal species such as *Candida albicans* and reduced levels of *Saccharomyces cerevisiae* have been reported, along with expansion of bacteriophages from the Caudovirales order in Crohn’s disease, highlighting the complexity of host–microbiome interactions in IBD [22,23].

## 4. Gut Metabolome in IBD

Metabolomics refers to the comprehensive and quantitative analysis of small-molecule metabolites produced by biological systems [24]. It provides a less invasive and highly sensitive approach to characterizing metabolites derived from both microbial communities and host cells across multiple biological matrices, including blood, stool, urine, and tissue samples [25].

Experimental studies using humanized mouse models, generated through transplantation of human fecal microbiota into germ-free mice, have demonstrated distinct metabolomic profiles when compared to those of conventionally raised mice. These findings indicate that differences in gut microbial composition can drive substantial alterations in host metabolic outputs [26,27].

Metagenomic and metabolomic investigations have shown that metabolites produced by diverse microbial communities can significantly influence host physiology and disease susceptibility. In murine models of dextran sulfate sodium (DSS)-induced colitis, marked metabolic alterations have been observed, including changes in tricarboxylic acid (TCA) cycle intermediates and amino acid profiles in both serum and colonic tissues. These metabolic shifts correlated with disease severity, and glutamine supplementation was shown to attenuate DSS-induced colitis, suggesting a potential therapeutic role for targeting microbial–host metabolic interactions [28,29].

### 4.1. Triglycerides

Triglycerides consist of fatty acids, which are classified into short-chain, medium-chain, and long-chain fatty acids [30]. Among these, short-chain fatty acids (SCFAs), primarily acetate, propionate, and butyrate; are produced through the bacterial fermentation of indigestible fibers in the intestine and play a key role in intestinal homeostasis [31]. Several studies have reported reduced fecal levels of SCFAs in patients with IBD, with lower concentrations of acetate and butyrate compared with healthy controls [30].

Beyond their metabolic function, SCFAs exert important immunomodulatory effects. Experimental evidence indicates that SCFAs can suppress intestinal inflammation by modulating immune pathways, including the induction of interleukin-22 (IL-22), which contributes to epithelial protection [32]. In addition, reduced levels of medium-chain fatty acids, such as pentanoate, hexanoate, heptanoate, and octanoate, have been described in IBD patients and have been associated with anti-inflammatory properties, supporting their potential therapeutic relevance [33,34].

### 4.2. Amino Acids

Alterations in amino acid metabolism are another characteristic feature of IBD. Patients exhibit reduced metabolism of several amino acids, which may contribute to intestinal inflammation and barrier dysfunction. Dietary tryptophan deficiency has been associated with exacerbation of colitis, whereas supplementation with tryptophan and its microbial-derived metabolites has been shown to ameliorate dysbiosis, reduce inflammation, and preserve epithelial barrier integrity [35,36].

Similarly, lower arginine levels have been observed in IBD patients, and experimental models suggest that impaired arginine catabolism—such as in the absence of arginase 1—is associated with more severe colitis [37]. These findings highlight amino acid metabolism as a potential therapeutic target in IBD.

### 4.3. Secondary Bile Acids

Bile acid metabolism is markedly altered in IBD, with increased levels of primary bile acids and a concomitant reduction in secondary bile acids. These changes have been linked to both microbial dysbiosis and intestinal inflammation. Experimental studies have shown that a shift toward primary bile acid enrichment can promote gut inflammation through microbiota-dependent mechanisms, whereas secondary bile acids—particularly deoxycholic acid (DCA)—exert anti-inflammatory effects and support intestinal barrier integrity [38,39,40].

Moreover, a recent study highlighted the correlation between a reduction in secondary bile acids and an increase in beneficial bacterial species such as *Roseburia*, *Clostridium IV*, *Butyricicoccus*, and *Faecalibacterium* in UC patients, suggesting a role for secondary bile acids in shaping microbial diversity [41].

Secondary bile acids have also been linked to the activation of the farnesoid X receptor (FXR), a nuclear receptor that regulates gut homeostasis and immune responses. Dysregulation of FXR signaling in IBD may therefore represent a mechanistic link between bile acid imbalance, microbial alterations, and intestinal inflammation [42].

Restoring a balanced bile acid profile may thus offer novel therapeutic opportunities in IBD.

### 4.4. Linking Metabolomic Alterations to Drug Metabolism and Therapeutic Response

The metabolomic alterations described above—including reduced short-chain fatty acids, altered amino acid metabolism, and imbalanced bile acid profiles—have direct implications for understanding drug–microbiota interactions in inflammatory bowel disease (IBD) [43].

Microbial communities responsible for producing beneficial metabolites, such as butyrate, also harbor a wide range of enzymatic activities capable of transforming xenobiotics and pharmacological compounds [44].

For instance, the gut microbiota can enzymatically activate certain IBD prodrugs (e.g., sulfasalazine) while also participating in the metabolism or inactivation of other therapeutic agents through microbial enzymatic pathways [44,45].

Furthermore, the metabolic capacity of the dysbiotic microbiome observed in patients with IBD differs substantially from that of healthy individuals. Patients with severe disease often exhibit reduced microbial diversity and depletion of short-chain fatty acid-producing bacteria, suggesting that microbial drug metabolism may be altered in the context of active intestinal inflammation [46].

These observations indicate that microbial metabolic pathways may represent an important mechanistic link between microbiome composition and variability in therapeutic response. This metabolic framework provides the conceptual basis for understanding how specific therapeutic classes interact with the gut microbiome, as discussed in the following section.

## 5. Gut Microbiota and Drug Metabolism

One of the major unresolved challenges in IBD management is the marked interindividual variability in response to available therapies. Increasing evidence suggests that the intestinal microbiota plays a relevant role in modulating this variability [47,48].

Notably, different intestinal compartments appear to contribute distinctly to drug metabolism: the duodenal microbiota is primarily involved in the metabolism of systemically administered drugs, such as corticosteroids and azathioprine, whereas the colonic microbiota predominantly affects locally acting agents, including mesalamine [49]. In addition, indirect microbiota–drug interactions may occur through alterations in bile acid metabolism.

Several studies have shown that anti-Tumor Necrosis Factor (TNF)-alpha therapy, particularly infliximab, is associated with changes in microbial composition and diversity. Treatment has been linked to increased fecal microbial diversity, enrichment of short-chain fatty acid-producing taxa (e.g., *Roseburia*, *Lachnospira*, *Blautia*), and a reduction in potentially pathogenic bacteria such as *Fusobacterium* and Enterobacteriaceae [50,51]. Notably, anti-TNF therapy has been associated with divergent effects on fecal and mucosal microbiota, with increased fecal microbial diversity but reduced diversity in mucosal biopsy samples, suggesting compartment-specific microbiota–drug interactions. Conversely, thiopurine exposure has been associated with reduced fecal microbial diversity in some cohorts [52]. Whether these shifts reflect direct drug–microbiota interactions or secondary effects of inflammation control remains unclear.

The intestinal microbiota plays a critical role in the activation and metabolism of aminosalicylates. Sulfasalazine requires bacterial azoreductases, produced by species such as *Escherichia coli*, *Enterococcus faecalis*, and *Pseudomonas aeruginosa*, to cleave its azo bond and release the active moiety in the intestinal lumen [53]. Interindividual variability in microbiota composition has been shown to influence sulfasalazine degradation rates in ex vivo fecal cultures, supporting a potential role for microbiota-driven variability in treatment efficacy [54]. In addition, bacterial N-acetyltransferase 1 activity contributes to the inactivation of 5-aminosalicylic acid (5-ASA), potentially affecting therapeutic response [55]. Beyond metabolic activation and inactivation, 5-ASA has been shown to interfere with bacterial biofilm formation and to inhibit the growth of selected pathogenic taxa, including *Escherichia coli* and *Campylobacter concisus*, while the sulfapyridine moiety of sulfasalazine exhibits antibiotic activity against members of the Enterobacteriaceae family [56,57,58]. These dual effects suggest a potential role for aminosalicylates in partially correcting dysbiosis, although their clinical relevance remains to be fully established.

Corticosteroid–microbiota interactions represent a less explored area. Several intestinal bacterial strains, including *Bacteroides eggerthii* and *Clostridium scindens*, have been shown to degrade corticosteroids such as budesonide and prednisone in vitro, potentially influencing drug bioavailability [59,60]. Proposed mechanisms include bacterial steroid-12,20-desmolase activity; however, its limited expression among intestinal bacteria suggests the involvement of alternative or complementary metabolic pathways. While in vitro studies have reported minimal effects of corticosteroids on bacterial growth, animal and human studies suggest that corticosteroid therapy may alter microbial composition and diversity, with differences observed between responders and non-responders [61,62,63,64,65,66]. These inconsistencies highlight the need for longitudinal, well-controlled studies to clarify the clinical impact of microbial corticosteroid metabolism in IBD.

The intestinal microbiota may also contribute to thiopurine metabolism. In vitro and animal studies have identified bacterial strains capable of converting azathioprine and 6-thioguanine into active metabolites, although no single species appears to possess the full enzymatic machinery required for complete conversion [67,68]. Importantly, *Bacteroides fragilis ATCC 25285* lacks glutathione S-transferase, a key enzyme for the conversion of azathioprine to 6-mercaptopurine, suggesting that cooperative microbial networks may be required for efficient thiopurine metabolism. However, clinical evidence supporting a direct impact of microbiota-mediated thiopurine conversion on treatment outcomes remains limited, warranting cautious interpretation.

Certain bacterial enzymes, such as immunoglobulin-degrading proteases, have been shown in vitro to modify monoclonal antibodies. In particular, IdeS produced by *Streptococcus pyogenes* can cleave IgG and may potentially alter the effector function of monoclonal antibodies, including infliximab [69,70,71,72]. The extent to which such mechanisms operate in vivo in IBD patients remains unclear.

Data on small-molecule therapies is scarce. While most tofacitinib is absorbed systemically, unabsorbed drug and metabolites may interact with the intestinal microbiota. Experimental data suggest that tofacitinib can alter microbial composition in murine models, although direct microbiota–drug interactions remain speculative given the mammalian specificity of Janus kinase (JAK) signaling [69,73]. However, as tofacitinib targets the ATP-binding site of JAKs, off-target interactions with structurally homologous microbial proteins cannot be excluded. Further studies integrating in vitro models, fecal cultures, and clinical cohorts are needed to clarify the impact of small molecules on microbiota composition and function and their relevance to therapeutic response.

### Small-Molecule Therapies and the Gut Microbiota

Among pharmacological therapies used in IBD, small-molecule drugs represent an emerging class whose interaction with the gut microbiota may differ substantially depending on their pharmacological targets and metabolic pathways, with important implications for understanding their mechanisms of action [74,75].

JAK inhibitors, including tofacitinib, upadacitinib, and filgotinib, exert their therapeutic effects through intracellular inhibition of the JAK-STAT signaling pathway [74].

Given that JAK-STAT signaling is specific to mammalian cells and that these drugs are rapidly absorbed systemically, their capacity for direct interaction with gut bacteria is inherently limited. Recent experimental evidence supports this prediction: studies using calorimetric measurements, optical density assessments, and metagenomic sequencing demonstrated that JAK inhibitors have a minor impact on the composition and function of human gut microbiota, with tofacitinib showing no significant effect on microbiota composition, fermentative activity, or metabolic output after 48 h of treatment [75,76]. These findings indicate that any observed microbial changes during JAK inhibitor therapy likely represent indirect effects mediated by the reduction in intestinal inflammation rather than direct pharmacological interactions with bacterial communities.

In contrast, S1P receptor modulators demonstrate direct and bidirectional interactions with the gut microbiota. Ozanimod exerts significant antimicrobial effects, leading to marked alterations in microbial composition characterized by enrichment of the *Enterococcus* genus and a substantially different metabolic landscape compared to untreated microbiota [76].

## 6. Microbial Predictors

### 6.1. Microbial Predictors of Anti-TNFalpha Therapy Response

Several studies have investigated the role of gut microbiota as predictors of response TNF-α inhibitors in IBD. In CD, responders to infliximab (IFX) consistently exhibit higher baseline microbial diversity and richness compared with non-responders [51]. At the phylum level, Firmicutes have emerged as key predictors of therapeutic response. Higher baseline and post-treatment abundance of Firmicutes has been associated with improved response to anti-TNF-α therapy in both UC and CD. Within this phylum, several taxa have been recurrently linked to favorable outcomes, including *Clostridium colinum*, *Eubacterium rectale*, *Faecalibacterium prausnitzii*, members of the Clostridia class, and families such as Ruminococcaceae and Anaerosporobacter [77,78].

Consistently, Lee et al. reported that increased abundance of *Faecalibacterium prausnitzii* and *Ruminococcus bromii* was associated with clinical remission at weeks 14 and 52, as well as endoscopic remission, irrespective of IBD subtype. In contrast, higher abundance of taxa such as *Bacteroides ovatus*, *B. thetaiotaomicron*, and *Veillonella parvula* was observed in non-responders. Interestingly, this study also identified therapy-specific microbial signatures: *Clostridium citroniae* and *Anaerobutyricum butyriciproducens* were associated with remission under anti-TNF-α and anti-IL-12/23 therapy, whereas *Bacteroides stercoris* was linked to response to anti-integrin agents. Other taxa, including *Bacteroides caccae* and *B. ovatus*, were associated with remission regardless of treatment class [79]. Highlighting the predictive value of microbial profiling, Busquets et al. developed an algorithm based on four bacterial markers—including *F. prausnitzii*—that accurately discriminated responders from non-responders to anti-TNF-α therapy in a cohort of UC and CD patients. High abundance of *F. prausnitzii* before and after treatment was strongly predictive of clinical remission [80]. In CD, increased abundance of *Clostridiales* has also been associated with response to infliximab, predicting treatment efficacy with an accuracy of 86.5%, which increased to 93.8% when combined with fecal calprotectin levels [81]. Beyond Firmicutes, responders to anti-TNF-α therapy have been shown to display increased abundance of Bacteroidetes and reduced levels of Proteobacteria, both at baseline and following treatment [78,82]. Supporting these observations, Höyhtyä et al. demonstrated that pediatric responders (both UC and CD) exhibited higher baseline abundance of the Bifidobacteriales order, consistent with findings in adult CD cohorts [83]. Similarly, Park et al. reported that greater clinical response was associated with lower Proteobacteria levels, which further decreased after treatment in responders [82]. In contrast, non-responders showed increased abundance of *Fusobacterium*, a genus associated with adverse inflammatory outcomes [84].

Finally, microbial metabolites also appear to contribute to therapeutic response. SCFAs, particularly butyrate, and metabolic pathways involved in bile acid synthesis have been linked to treatment efficacy. Higher levels of butyrate and substrates involved in its synthesis, such as ethanol and acetaldehyde, were significantly associated with clinical remission following anti-TNF-α therapy [85].

### 6.2. Effects of Anti-TNF Alpha on Gut Microbiota

Significant changes in gut microbial diversity have been observed in patients with CD undergoing anti-TNF-α therapy. Treatment has been associated with increased β-diversity compared with pre-treatment profiles [86]. In responders, an increase in α-diversity has been consistently reported after six months of therapy, suggesting a shift toward a more eubiotic microbiome [87,88].

Seong et al. showed that mucosal healing during infliximab therapy was associated with greater microbial diversity and with infliximab trough levels (TLI) ≥5 μg/mL. Consistently, TLI ≥ 5 μg/mL and mucosal healing were associated with increased abundances of *F. prausnitzii* and *Blautia* [89].

Anti-TNF-α therapy appears to actively modulate gut microbiota composition. Responders show increased abundance of Firmicutes during treatment, particularly genera such as *Lactococcus*, *Roseburia*, and *F. prausnitzii*, whereas lower Firmicutes levels have been reported in patients experiencing relapse after infliximab therapy [90,91]. These effects may be partially mediated by enrichment of SCFA-producing taxa, contributing to inflammation control and restoration of bile acid metabolism [92,93]. In pediatric CD, sustained response to infliximab (PCDAI ≤ 10) has similarly been associated with expansion of SCFA-producing genera, including *Blautia*, *Faecalibacterium*, *Lachnospira*, and *Roseburia* [93].

In addition, increased abundance of *Ruminococcus* has been repeatedly associated with favorable response to biologic therapy, supporting its role as a potential microbial marker of response [92,94,95]. From an immunological perspective, anti-TNF-α therapy has been associated with increased IgM levels and expansion of pre-switch memory B cells, suggesting that microbiota composition may influence therapy-induced immune modulation [92].

Reduction in Proteobacteria also appears to be a consistent feature of treatment response. Longitudinal studies have shown a decrease in Proteobacteria in responders, whereas persistence of high Proteobacteria levels—particularly Enterobacteriaceae and *Escherichia/Shigella*—has been associated with treatment resistance [96,97]. The *F. prausnitzii/E. coli* ratio has been proposed as a reliable predictor of response to anti-TNF-α therapy in CD, with an area under the curve of 0.87 [88].

Finally, microbial metabolites have emerged as additional predictors of therapeutic response. Non-responders exhibit higher levels of sulphate- and glycine-conjugated primary bile acids, whereas increased levels of secondary bile acids, particularly deoxycholic acid, have been associated with response to anti-TNF-α therapy [98]. Moreover, higher levels of butyrate and its metabolic substrates (ethanol and acetaldehyde) have been linked to clinical remission, while impaired metabolite exchange between bacterial communities has been observed in non-responders [85].

### 6.3. Microbial Predictors of Anti-Integrin α4β7 and Anti-Interleukin-12/23 Therapies

Compared with TNF-α inhibitors, data on microbial predictors of response to vedolizumab (VDZ) and ustekinumab (UST) are more limited. For VDZ, Ananthakrishnan et al. reported that patients achieving clinical remission exhibited higher baseline α-diversity and greater abundance of *Roseburia inulinivorans*, *Burkholderiales* species, and pathways related to branched-chain amino acid biosynthesis compared with non-responders. Importantly, a microbiota-based predictive model showed superior performance (AUC = 0.715) compared with clinical variables alone (area under the curve (AUC) = 0.619). Baseline abundance of several anti-inflammatory taxa, including *Phascolarctobacterium faecium*, *Agathobaculum butyriciproducens*, and *Clostridium citroniae*, was also associated with earlier remission [99].

Consistent findings were reported by Colman et al. in a cohort of CD and UC patients, in whom higher baseline abundance of the butyrate-producing *Anaerostipes hadrus* was associated with therapeutic VDZ through concentrations and improved clinical response at week 14. An even stronger predictive signal was observed at week 2, where *A. hadrus,* together with other butyrate-producing Firmicutes, including *Faecalibacterium prausnitzii*, predicted therapeutic drug levels at week 14 [100].

Beyond microbial composition, metabolomic signatures have also been implicated. In a cohort of UC patients, Jiang et al. demonstrated that baseline metabolite profiles assessed by gas chromatography–mass spectrometry (GC-MS) predicted early clinical remission at week 14. A combined model including acetamide, taurine, and putrescine showed strong prognostic performance in patients with moderate-to-severe UC treated with vedolizumab [101].

Data on microbial predictors during ustekinumab therapy remain scarce. Multi-omics analyses have identified microbial and metabolic profiles associated with remission under anti-cytokine therapies; however, these studies often included mixed treatment groups, limiting conclusions specific to ustekinumab [79].

### 6.4. Effects of Anti-Integrin α4β7 and Anti-Interleukin-12/23 Therapies on Gut Microbiota

In UC patients treated with vedolizumab, an enrichment of bacteria with anti-inflammatory properties, including *Bifidobacterium longum* and *Bacteroides sartorii*, has been reported [101,102]. Similarly, in mixed IBD cohorts, remission and therapeutic VDZ concentrations were associated with increased abundance of butyrate-producing species and enrichment of microbial pathways involved in butyrate synthesis [99,100].

In CD patients refractory to anti-TNF-α therapy, Doherty et al. observed that responders to ustekinumab displayed higher microbial diversity compared with non-responders. Patients in remission six weeks after induction showed increased abundance of Firmicutes—particularly *Ruminococcaceae*, *Faecalibacterium*, *Blautia*, *Clostridium* cluster XIVa, and *Roseburia*—whereas patients with active disease exhibited higher relative abundance of *Escherichia/Shigella* belonging to the phylum Proteobacteria [103].

### 6.5. Impact of Non-Biologic Therapies on Gut Microbiota

Non-biologic therapies also exert measurable effects on gut microbiota composition. Treatment with 5-ASA has been associated with increased α-diversity as early as 14 days after initiation [104]. Corticosteroid therapy has similarly been linked to a significant increase in α-diversity after 12 weeks of treatment [105].

In contrast, antibiotic exposure is consistently associated with reduced microbial diversity. In patients with Crohn’s disease, regimens including metronidazole alone or in combination with azithromycin were associated with a marked decrease in α-diversity during the first 8 weeks of treatment, with persistent effects observed over a 12-week period.

**Figure 1:**
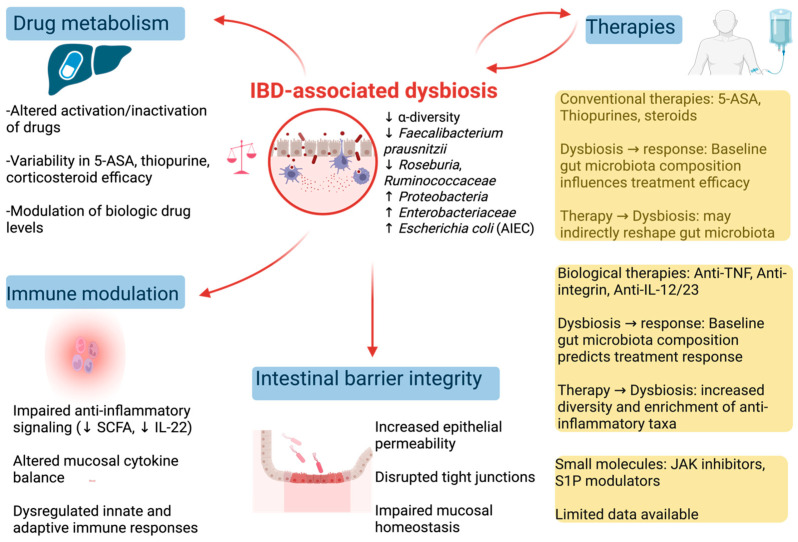
IBD-associated dysbiosis and its effect in IBD pathogenesis and therapeutic outcomes. IBD-associated dysbiosis affects drug metabolism, immune function, and barrier integrity. Gut microbiota composition influences response to conventional and biological therapies, which in turn can modulate microbial diversity and function.

**Figure 2:**
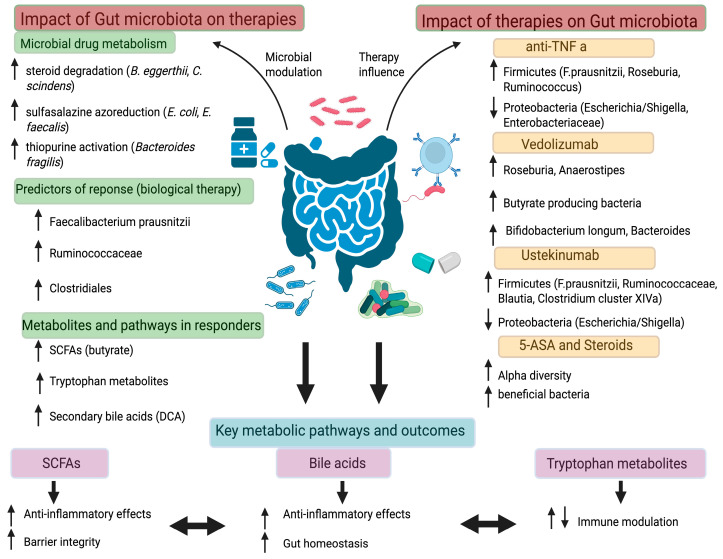
Bidirectional interactions between gut microbiota and therapeutic agents in IBD. Left panel: microbial taxa influence drug metabolism, efficacy, and pharmacokinetics. Right panel: the effects of therapeutic interventions on gut microbial composition, highlighting taxa that are enriched or depleted. Bottom panel: key metabolic pathways and functional outcomes, including short-chain fatty acid production, bile acid transformation, and modulation of inflammatory responses, linking microbial shifts to clinical and pharmacological consequences.

The bidirectional interactions between gut microbiota and therapeutic interventions in IBD are illustrated in Figure 1 and Figure 2. Detailed therapy-associated microbiota changes, microbial predictors of response, and key metabolites are summarized in Table 1 and Table 2.

## 7. Discussion and Future Perspectives

The intestinal microbiota represents a promising frontier in optimizing therapeutic strategies for IBD. However, current research is subject to several limitations.

Interpretation of microbiota—multiple layers of confounding and marked methodological heterogeneity further complicate treatment associations. Variability in sequencing approaches (16S rRNA gene sequencing versus shotgun metagenomics), bioinformatic pipelines, reference database, and diversity metrics limits cross-study comparability. Another major limitation concerns the definition of treatment response, as many studies rely primarily on clinical remission without incorporating endoscopic healing, which represents a key surrogate of long-term disease control. Notably, Lee et al. demonstrated that both clinical and endoscopic remission correlate with an increased abundance of *Faecalibacterium prausnitzii* and *Ruminococcus bromii*, suggesting that incorporating mucosal healing as an endpoint in future studies could enhance the identification of microbial biomarkers predictive of therapeutic outcomes.

A key unresolved question is whether dysbiosis drives therapeutic failure or merely reflects persistent inflammation. Evidence from germ-free models supports a causal role: transplantation of IBD-derived microbiota into germ-free mice increases intestinal Th17 cells, reduces RORγt+ regulatory T cells, and exacerbates colitis compared to healthy donor microbiota [106,107].

Furthermore, disease-associated microbial communities have been reported to transmit Crohn’s disease-like ileitis to genetically susceptible germ-free recipients [108]. However, the relationship is likely bidirectional, with intestinal inflammation itself reshaping microbial ecosystems and further amplifying dysbiosis [109].

This framework has important implications: baseline microbial signatures may represent true predictive biomarkers, whereas changes observed during treatment may reflect inflammation control rather than direct drug effects. Future studies with clearly defined sampling timepoints will be essential to distinguish these scenarios.

Taken together, the available evidence suggests that microbiota-related mechanisms influencing therapeutic response in IBD should not be interpreted as isolated pathways but rather as complementary mechanisms. Microbial composition determines the production of metabolites such as SCFAs, amino acid derivatives, and secondary bile acids, which collectively regulate epithelial barrier integrity, mucosal immune homeostasis, and inflammatory pathways. These metabolic products may indirectly influence therapeutic efficacy by modifying the inflammatory environment in which drugs act. At the same time, specific bacterial taxa can directly influence drug bioavailability by enzymatically activating, degrading, or modifying several compounds, including aminosalicylates, corticosteroids, and thiopurines. Conversely, pharmacological treatments can induce shifts in microbial composition, thereby influencing microbial metabolism and host immune responses. Considering the marked interindividual differences in gut microbial composition and metabolic potential, these interconnected mechanisms may underline variability in treatment response among patients receiving the same therapy.

Building on this mechanistic understanding, microbiome-based predictive models have emerged as a promising tool for precision medicine in IBD. By integrating microbial composition, diversity, and metabolomic profiles, these models can stratify patients by predicted response to therapies such as anti-TNF, anti-integrin, or anti-IL-12/23 agents. In a proof-of-concept study, stool samples from 38 IBD patients initiating anti-TNF therapy (adalimumab, golimumab, or infliximab) were analyzed to quantify eight microbial markers. Patients were classified as responders or non-responders, and an algorithm combining four bacterial taxa was developed. This model distinguished responders from non-responders with 93.3% sensitivity, 100% specificity, a positive predictive value of 100%, and a negative predictive value of 75%, suggesting that a defined microbial signature could help guide personalized anti-TNF therapy [80].

Conversely, data on JAK inhibitors remain limited. Given their intracellular mechanism of action and rapid systemic absorption, direct interaction with the intestinal microbiota is uncertain, although modulation of immune signaling pathways may indirectly influence microbial composition.

Beyond biomarker discovery, a deeper understanding of microbiota-derived signatures associated with therapeutic response could facilitate the development of personalized treatment approaches. Identifying microbial predictors of drug efficacy may enable a more tailored selection of therapeutic strategies, optimizing patient outcomes. Moreover, the advancement of next-generation probiotics and microbiome-targeted interventions holds significant promise. These strategies may restore microbial homeostasis, enhance anti-inflammatory pathways, or modulate drug metabolism, ultimately improving therapeutic efficacy while reducing adverse effects. Emerging multi-omics technologies, including metagenomics, metabolomics, and transcriptomics, will be instrumental in elucidating the intricate interplay between the gut microbiota, host immunity, and treatment response.

An important limitation of the current literature—and, consequently, of this review—is the limited consideration of dietary interventions, herbal compounds, and complementary therapies as potential confounding variables in microbiome–drug interaction studies.

Diet is increasingly recognized as a critical modulator of gut microbiota composition and function in IBD, with Mediterranean-style diets promoting favorable microbiome modifications characterized by the enrichment of butyrate-producing taxa such as *Faecalibacterium prausnitzii* and *Roseburia* species [110,111]. Furthermore, complementary and alternative medicines—including curcumin, cannabis-derived products, and various herbal preparations—are frequently used by patients with IBD and may independently influence microbial composition and metabolic activity [112,113].

Their concurrent use alongside pharmacological therapies therefore represents a potential confounding factor that is rarely controlled for in existing studies investigating microbiome–drug interactions. Future pharmacomicrobiomic investigations should systematically account for dietary patterns and complementary therapies to improve the interpretability and generalizability of findings.

While this review provides a comprehensive synthesis of available evidence, the narrative approach inherently limits the formal strength of the conclusions due to potential selection bias and heterogeneity across study design, sequencing methods, and outcome definitions. Nonetheless, this format allows the integration of diverse evidence, highlights consistent microbial trends, identifies emerging predictive signatures, and outlines knowledge gaps that can guide future research and inform microbiota-informed therapeutic strategies. Looking ahead, integrating microbiota analysis into clinical practice has the potential to transform IBD management, shifting from an empirical approach to a precision-medicine paradigm. Large-scale, longitudinal studies are essential to validate microbial biomarkers and assess their clinical utility. Ultimately, leveraging the therapeutic potential of the microbiota may not only enhance disease management but also provide novel insights into the pathogenesis and progression of IBD, paving the way for innovative therapeutic strategies.

## Figures and Tables

**Table 1:** The impact of therapy on gut microbiota. Table 1 summarizes the main effects of commonly used IBD therapies on gut microbiota composition and diversity, highlighting treatment-associated changes in specific bacterial taxa and overall microbial profiles.

Therapeutic Tool	Microbiota Modulation	Reference	Results Summary
5-ASA	*<Escherichia coli (*UPEC*)* and *P. aeruginosa*	Dahl, J.U. et al. [50]	- 5-ASA decrease polyphosphate levels, sensitizes bacteria towards oxidative stress, reduces colonization and attenuates cell and biofilm formation.
5-ASA	>*Faecalibacterium prausnitzii*, *Blautia*, *Bacteroides*	Olaisen, M. et al. [51]	- 5-ASA increase bacterial diversity within the mucosa, increasing beneficial species.
5-ASA	*<E. coli* and *Campylobacter concisus*	Liu, F. et al. [52]	- 5-ASA in vitro analyses inhibit the growth of pathogenic bacteria.
Corticosteroids		Maier, L. et al. [55]	- No significant changes in bacterial growth across 40 intestinal strains following CS administration.
Corticosteroids	*<Bifidobacterium longum*	Li, L. et al. [58]	- CS may influence specific bacterial populations.
Corticosteroids	>*Actinomyces*	Schrirmer, M. et al. [60]	- CS are significantly associated with changes in microbial abundance.
Thiopurine	>Firmicutes and <Bacteroidetes	Oancea, I. et al. [62]	- 6-thioguanine suggests a potential microbiota-modulating effect.
Thiopurine		Wills, E.S. et al. [46]	- Azathioprine and 6-MP resulted in a reduction in microbial richness and overall community diversity.
Anti-TNF-a (IFX)	>*Roseburia*, *Lachnospira*, *Blautia;* *<Fusobacterium*, *Enterobacter*, *Escherichia*	Dovrolis, N. et al. [44] Zhuang, X. et al. [45]	- IFX increases fecal microbial diversity, promoting increase in SCFA-producing bacteria while reducing opportunistic pathogenic bacteria.
Anti-TNF-a		Dovrolis, N. et al. [44]	- IFX has been associated with increased fecal microbiota diversity and shifts in specific bacterial taxa.
Anti-TNF-a	>*F. prausnitzii* and *Blautia*	Seong, G. et al. [80]	- IFX-induced mucosal healing was associated with greater diversity of gut microbiota.
Anti-integrine a4b7	>*Bifidobacterium longum* and *Bacteroides sartorii*	Jiang, L. et al. [92] Sharma, S. et al. [93]	- UC patients during VDZ therapy have an increase in bacteria with anti-inflammatory capabilities.
Anti IL12/23	>Ruminococcaceae, *Faecalibacterium*, *Blautia*, Clostridium XIVa, *Roseburia* *<Escherichia/Shigella*	Doherty, M. K. et al. [94]	- Responders to Ustekinumab had an increased abundance of phylum Firmicutes and a reduced abundance of phylum Proteobacteria.

**Table 2:** Microbial predictors for therapy. Table 2 provides an overview of microbial features and specific bacterial taxa that have been associated with therapeutic response or non-response across different IBD treatment classes.

Therapy	Microbial Predictors	Reference	-
Corticosteroids	*Bacteroides eggerthii* and *Clostridium scindens*	Yadav, V. et al. [53]	- Various bacterial strains degrade CS, reducing their clinical efficacy.
Anti-TNF-a	>Firmicutes	Estevinho, M.M et al. [66]	- Higher levels of Firmicutes may be associated with improved response to anti-TNF therapy.
Anti-TNF-a	>*Clostridium colinum*, *Eubacterium rectale*, *F. prausnitzii*, *Clostridia*, Ruminococcaceae, *Anaerosporobacter*	Magnusson, M. et al. [68] Höyhtyä, M. et al. [69]	- A higher abundance of Firmicutes is more strongly associated with infliximab therapy in patients with UC.
Anti-TNF-a, anti IL12/23, anti-integrin **α4β7**	>*C. citronae*, *A. butyciproducens*, *B. stercoris*	Lee, J. et al. [70]	- The abundance of specific bacteria was associated with remission with anti-TNFa, anti-IL-12/23, anti-integrin **α4β7**.
Anti-TNF-a	>*F. prausnitzii*	Busquets, D. et al. [71]	- *F. prausnitzii* species counts predict good therapeutic efficacy and clinical remission.
Anti-TNF-a	>*Clostridiales*	Zhou, Y. et al. [72]	- Clostridiales abundance predicts 86.5% accuracy of treatment efficacy.
Anti-TNF-a	<Proteobacteria	Park, Y. E. et al. [73]	- Decreased levels of Proteobacteria were associated with greater clinical response.
Anti-TNF-a	>*Bifidobacteriales*	Zhang, Z. et al. [74]	- IFX pediatric CD responders demonstrated a higher abundance of Bifidobacteriales.
Anti-TNF-a	>*Lactobacillus*, *Roseburia*, *F. prausnitzii*	Rajca, S. et al. [82]	- Responders to anti-TNF exhibit an increase in the abundance of Firmicutes.
Anti-TNF-a	>*Blautia*, *Faecalibacterium*, *Lachnospira*, *Roseburia*	Wang, Y. et al. [84]	- In pediatric patients, the sustained response to IFX was positively associated with an expansion of SCFA-producing bacteria.
Anti-TNF-a	>*Ruminococcus* *<Proteobacteria*	Alatawi, H. et al. [85] Ribaldone, D. G. et al. [87]	- Responders to anti-TNF-a showed an increased abundance of *Ruminococcus* and a reduction in Proteobacteria.
Anti-TNF-a	>*Enterobacteria* (*Escherichia*/Shigella).	Sanchis-Artero, L. et al. [79]	- Association between treatment resistance and persistence of high concentration of Proteobacteria.
Anti-integrin a4b7	>*Roseburia inulinivorans* and Burkholderiales	Ananthakrishnan, A. et al. [90]	- Anti-integrin a4b7 responders have greater α-diversity and greater abundance of some species.
Anti-integrin a4b7	>*Anaerostipes hadrus*	Colman, R. J. et al. [91]	- Increase in butyrate-producing *Anaerostipes* was associated with a higher drug concentration at weeks 14 and better clinical response.

## Data Availability

No new data were created or analyzed in this study. Data sharing is not applicable to this article.

## Abbreviations

The following abbreviations are used in this manuscript:

IBD	Inflammatory Bowel Disease
FMT	Fecal Microbiota Transplantation
MeSH	Medical Subject Headings
DSS	Dextran Sulfate Sodium
TCA	Tricarboxylic Acid
CD	Crohn’s Disease
UC	Ulcerative Colitis
SCFAs	Short-Chain Fatty Acids
IL	Interleukin
GC-MS	Gas Chromatography–Mass Spectrometry
VDZ	Vedolizumab
UST	Ustekinumab
AUC	Area Under the Curve
TLI	Infliximab Trough Levels
PCDAI	Pediatric Crohn’s Disease Activity Index
IFX	Infliximab
JAK	Janus Kinase
5-ASA	5-Aminosalicylic Acid
TNF	Tumor Necrosis Factor
FXR	Farnesoid X Receptor
DCA	Deoxycholic Acid
